# Thermal Stabilization of an Endoglucanase by Cyclization

**DOI:** 10.1007/s12010-012-9674-z

**Published:** 2012-06-01

**Authors:** Johan F. T. van Lieshout, Odette N. Pérez Gutiérrez, Wietse Vroom, Antoni Planas, Willem M. de Vos, John van der Oost, Sotirios Koutsopoulos

**Affiliations:** 1 Laboratory of Microbiology, Wageningen University, Dreijenplein 10, 6703 HB Wageningen, the Netherlands; 2 Laboratory of Biochemistry, Institut Químic de Sarrià, Universitat Ramon Llull, Via Augusta 390, 08017 Barcelona, Spain; 3 Center for Biomedical Engineering, NE47-307, Massachusetts Institute of Technology, Cambridge, MA 02139-4307 USA

**Keywords:** Thermophilic enzyme, Circular protein, Enzyme stability, Cyclization, Thermostability

## Abstract

An intein-driven protein splicing approach allowed for the covalent linkage between the N- and C-termini of a polypeptide chain to create circular variants of the endo-β-1,3-1,4-glucanase, LicA, from *Bacillus licheniformis*. Two circular variants, LicA-C1 and LicA-C2, which have connecting loops of 20 and 14 amino acids, respectively, showed catalytic activities that are approximately two and three times higher, respectively, compared to that of the linear LicA (LicA-L1). The thermal stability of the circular variants was significantly increased compared to the linear form. Whereas the linear glucanase lost half of its activity after 3 min at 65 °C, the two circular variants have 6-fold (LicA-C1) and 16-fold (LicA-C2) increased half-life time of inactivation. In agreement with this, fluorescence spectroscopy and differential scanning calorimetry studies revealed that circular enzymes undergo structural changes at higher temperatures compared to that of the linear form. The effect of calcium on the conformational stability and function of the circular LicAs was also investigated, and we observed that the presence of calcium ions results in increased thermal stability. The impact of the length of the designed loops on thermal stability of the circular proteins is discussed, and it is suggested that cyclization may be an efficient strategy for the increased stability of proteins.

## Introduction

The application of enzymes in biotechnology and biocatalysis has increased considerably over the last few decades [1–4]. Desired features for optimal performance in these applications include high specificity, increased activity, and prolonged stability at elevated temperatures. Because mesophilic enzymes have evolved to function optimally at ambient temperatures, it is not surprising that such proteins do not meet most of the chemical, physical, and biological requirements for being ideal biocatalysts. Limited shelf life and low intrinsic stability are major drawbacks for the successful biotechnological application of mesophilic enzymes. Factors contributing to thermostability include ionic or hydrophobic interactions, improved subunit interactions, reduced surface area, increased packing, kinetic, thermodynamic and dynamic (including flexibility and rigidity) factors, or a combination thereof [5–9]. Both random and directed mutagenesis strategies have been used to create variants of mesophilic and thermophilic enzymes with increased stability features [10–13].

**Fig. 1:**
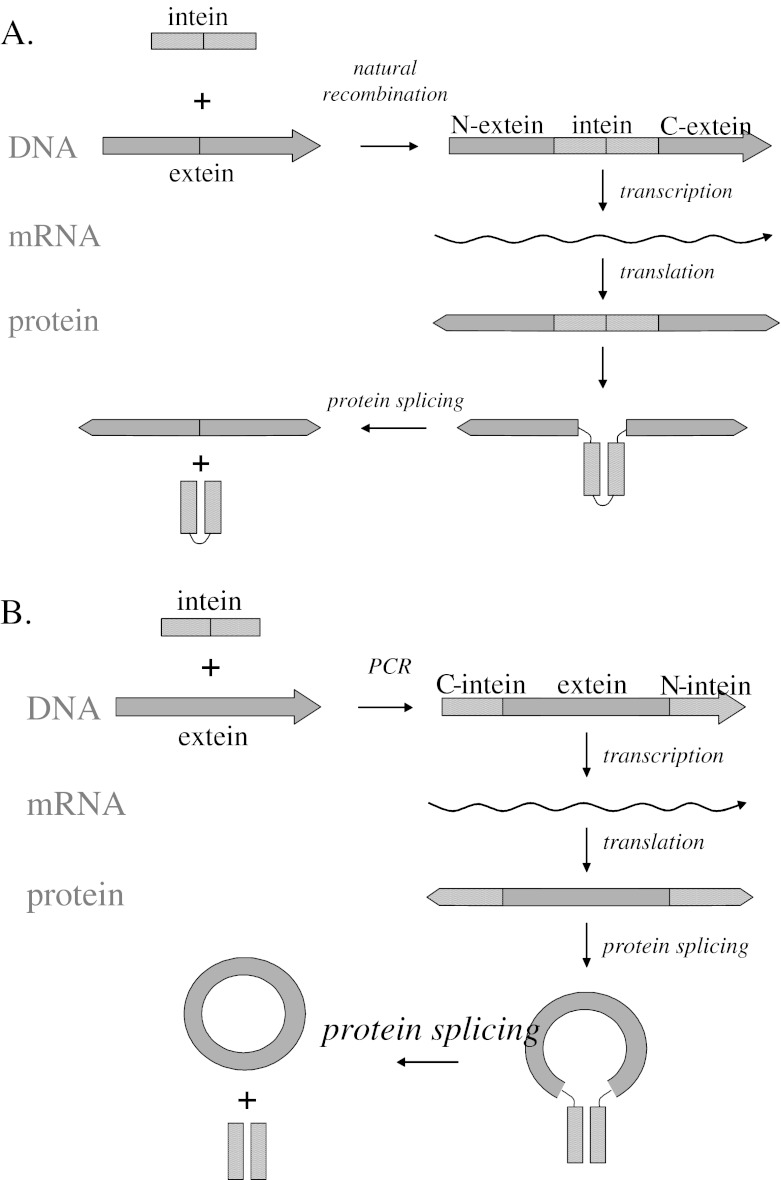
Inteins in nature and as tool in bioengineering. **a** Protein splicing by a natural intein, resulting in ligation of the two extein fragments. **b** The engineered split intein domains fold, interact, and form an active intein complex that catalyzes splicing and ligation of the extein fragment like the natural system. The result is a complex of the two intein domains and the extein domain with a circular peptide backbone

A poorly explored approach for the stabilization of a protein is the covalent cyclization of its backbone structure. Several naturally occurring peptides and small proteins have been identified in bacteria, plants, and mammals having covalently linked N- and C-termini [14–16]. It has been demonstrated that these circular peptides and proteins not only are more stable at a broad range of chemical and physical conditions but also often show an increased resistance to proteases [17]. Although details on the biosynthesis of naturally occurring circular proteins are largely unknown, several studies have shown that it is possible to engineer a covalent linkage between the N- and C-terminal amino acids of a polypeptide chain, thereby generating a circular enzyme. Several cyclization strategies have been described: (1) a chemical approach that employs solid phase synthesis combined with chemical ligation or cross-linking [18, 19]; (2) a biochemical strategy based on intein-driven self-ligation (*cis* and *trans*) in vitro [20–22] or on sortase-catalyzed transpeptidation [23]; and (3) a biological method that is based on the ability of inteins to perform self-splicing (*cis* and *trans*) in vivo (Fig. 1) [20, 24–27].

**Fig. 2:**
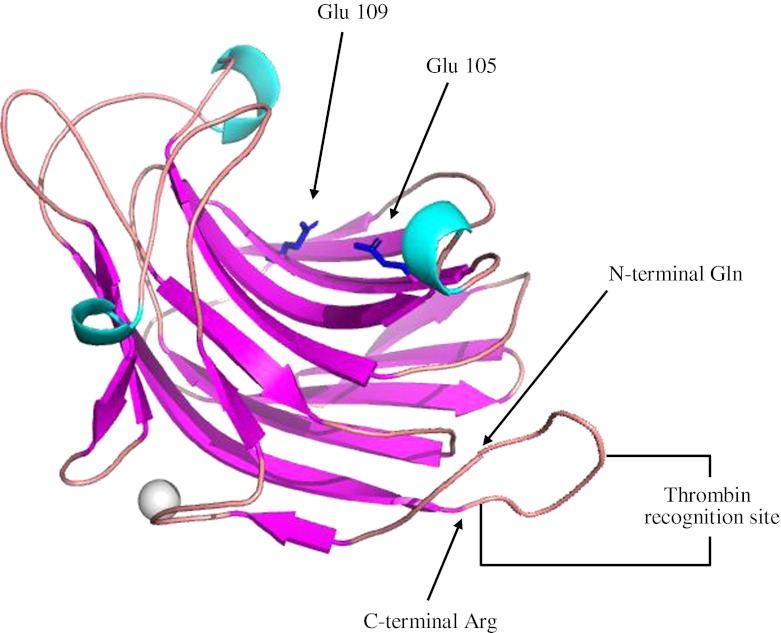
Circular LicA-C1 structural model in which 15 extra residues were added to the X-ray structure (Protein Data Bank code 1GBG) and manually turned to form a closed loop connecting the C- and N-termini. The thrombin recognition site (LVPRGT) is also shown. The two catalytic residues (*Glu-105* and *Glu-109*) are displayed in *blue*; the *gray sphere* marks the Ca^2+^ site

Bacterial 1,3-1,4-β-glucanases (or lichenases, EC 3.2.1.73) hydrolyze β-1,4-glycosidic bonds on 3-*O*-substituted glycosyl residues in linear mixed-linked glucans, like barley β-glucan and lichenan, and have been studied extensively over the years [28]. These enzymes are classified in family 16 of glycoside hydrolases [29] and have a monomeric jelly roll β-sandwich structure (Fig. 2) [30, 31].

In this study, we used a cyclization approach to covalently link the N- and C-termini of the thermophilic endo-β-1,3-1,4-glucanase (LicA) from *Bacillus licheniformis*. The crystal structure of LicA shows that the N- and C-termini are in close proximity, i.e., approximately 7 Ǻ, being part of the adjacent anti-parallel beta strands (Fig. 2) [30]. This structural property of LicA allows for employing the intein-based method of protein cyclization to generate a circular backbone structure. A number of circular LicA variants were produced and tested for their biological activity and stability at elevated temperatures.

## Materials and Methods

### Bacterial Hosts and Vectors

The T7 expression vector pET24d was obtained from Novagen. *Escherichia coli* XL-1 Blue (Stratagene) was used as an initial host for cloning, while the *E. coli* strains BL21(DE3) and JM109 (DE3) (Stratagene) were used as expression hosts for the pET derivatives. *E. coli* was grown in TY medium [32] in a rotary shaker at 37 °C. Kanamycin was added to a final concentration of 30 μg/ml.

### Cloning and Expression

The gene coding for *B. licheniformis* 1,3-1,4-β-glucanase previously cloned in the pUC119-derived pD6-2 [33] was used as a template for PCR amplification. For expression of the linear enzyme without the signal sequence, the gene was subcloned in pET24d using primers BG1306 and BG1307 (Table 1), introducing a C-terminal His-tag, resulting in pWUR146.

**Table 1:** Primer sequences used for the expression of the linear and circular LicA protein derivatives in *E. coli*

Primer^a^	Primer sequence^b^	Description	LicA variant
BG1260 s	GCGCGCCATGGGACATGAGTACATCTATGACAGA	Intein C + *Nco*I site	C1–C6, C1_control_
BG1261 a	GTGGTGGTGGTGGTGGTGTCCGGTGTTGTGGACGAAAATCATTC	Intein C + His-tag	C1, C2, C6, C1_control_
BG1262 s	GGACACCACCACCACCACCACCAAACGGGCGGGTCGTTTTATGAAC	Glucanase + His-tag	C1, C2, C6, C1_control_
BG1263 a	CCCGGTTCCTCGTGGTACTAGTCTTTTTGTGTAACGCACCCAATG	Glucanase + thrombin site	C1, C5, C1_control_
BG1264 s	CTAGTACCACGAGGAACCGGGTGCATAGACGGAAAGGCCAAG	Intein N + thrombin site	C1, C5, C1_control_
BG1265 a	GCGCGCTCGAGCTTAACATGTGAGTGGTATTTATC	Intein N + *Xho*I site	C1_control_
BG1306 s	GCGCGCCATGGGGCAAACGGGCGGGTCGTTTT	Glucanase + *Nco*I site + Δsignal seq + SA→MG	L1
BG1307 a	GCGCGCTCGAGTCTTTTTGTGTAACGCACCCA	Glucanase + *Xho*I site + Δstop codon	L1
BG1351 s	GGACACCACCACCACCACCACGGGTCGTTTTATGAACCGTTCAAC	Glucanase + His-tag + ΔQTG	C3, C4, C5
BG1352 a	TGGCCTTTCCGTCTATGCACCCTGTGTAACGCACCCAATGTAATGAG	Glucanase + thrombin site + Δthrombin site + ΔKR	C3, C6
BG1353 s	CTCATTACATTGGGTGCGTTACACAGGGTGCATAGACGGAAAGGCCA	Intein N + thrombin site + Δthrombin site + ΔKR	C3, C6
BG1354 a	GCGCGCTCGAGTTACTTAACATGTGAGTGGTATTTATCAAA	Intein N + *Xho*I site + stop codon	C1–C6
BG1429 a	TCTATGCACCCTCTTTTTGTGTAACGCACCCAATG	Glucanase + thrombin site + Δthrombin site	C2
BG1430 s	GTTACACAAAAAGAGGGTGCATAGACGGAAAGGCC	Intein N + thrombin site + Δthrombin site	C2
BG1431 a	CCCGGTTCCTCGTGGTACTAGTGTGTAACGCACCCAATGTAATG	Glucanase + thrombin site + ΔKR	C4
BG1432 s	CTAGTACCACGAGGAACCGGGTGCATAGACGGAAAGGCC	Intein N + thrombin site + ΔKR	C4
Intein-f a	CGAGCCGAGGACGTTCTACGATC	Forward sequence primer, annealing to intein	–
Intein-r s	GCTTGTATCTCTCGTACATCTCCTC	Reverse sequence primer, annealing to intein	–

^a^“s” in the primer denotes “sense” and “a” denotes “antisense”

^b^Sequences are given from 5′–3′

**Fig. 3:**
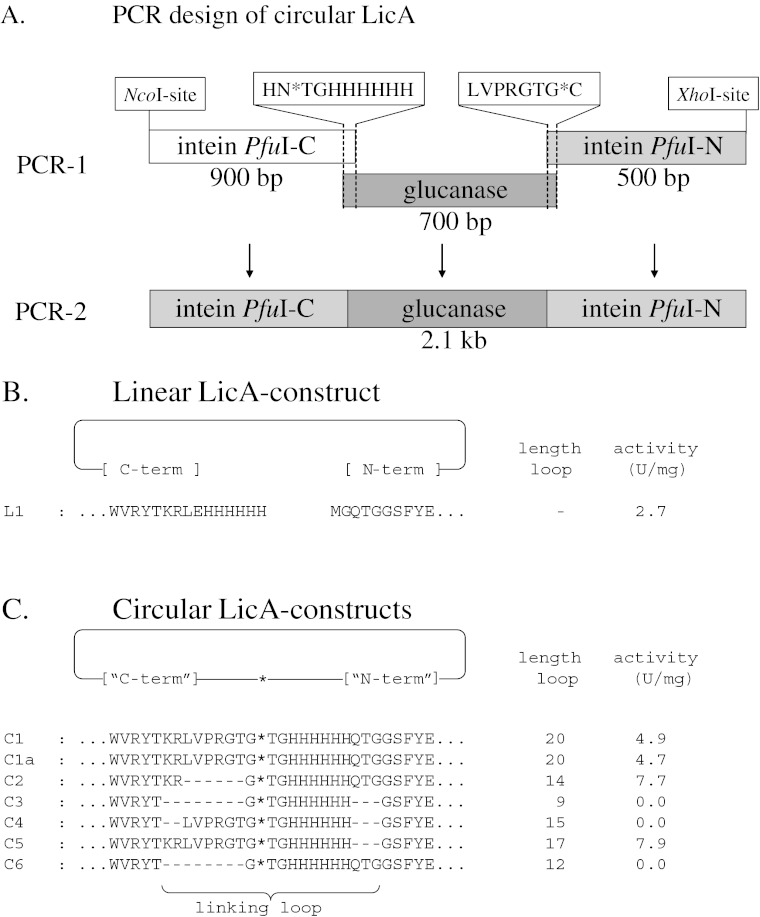
Circular constructs made in this study. **a** Schematic representation of the PCR-based engineering of the constructs used for the intein-based circularization of LicA. *NcoI* and *XhoI* are the introduced restriction sites. The amino acid sequence of the overlap of the PCR-1 fragments is shown. **b**, **c** Amino acid sequences of the N- and C-terminal regions of linear (**b**) and circular (**c**) variants of LicA. The extein sequences, corresponding to the wild-type sequence, are *underlined*. Note that in all constructs, the signal sequence has been deleted, resulting in intracellular production of the proteins. The length of the loops is given as well as the specific activity of the purified proteins (in units per milligram). *Asterisk* indicates the connection point of the N- and C-terminal sequence

*Pyrococcus furiosus* genomic DNA was isolated as described previously [32] and used as template for PCR amplification of the intein PI-PfuI [24, 34]. In a first series of PCR reactions, the two parts of the intein and the glucanase gene were amplified separately (Fig. 3a). In a subsequent PCR step, the three overlapping fragments were fused by overlap extension PCR [35], and the full-length hybrid molecule was amplified using the flanking primers BG1260 and BG1265/1354 (2.1 kb; Fig. 3a). The 2.1-kb PCR product was then ligated into pET24d at the *Nco*I and *Xho*I restriction sites and transformed to *E. coli* XL1-Blue. Sequence analysis of all variant constructs that were produced by this method (Table 1) was done by the dideoxynucleotide chain termination method with a Li-Cor automatic sequencing system (model 4000L). Two sets of sense/antisense sequence primers were used, which anneal either to the promoter and terminator sequence of the pET24d vector or to the sequences of the two parts of the intein flanking the glucanase gene (Intein-f and Intein-r in Table 1).

### Overexpression and Purification

Both the *licA-L1* gene and the permutated genes were expressed in freshly transformed BL21(DE3) or JM109 (DE3) cells. A 5-ml overnight culture was used to inoculate 500 ml of the TY medium containing 30 μg/ml kanamycin. When the OD_600_ reached 0.5, the cells were induced with 100 μM IPTG and subsequently grown at 37 °C for 4 h. Cells were harvested by centrifugation (10 min, 4,000×*g*) and resuspended in 50 mM sodium phosphate buffer (pH 7.7) containing 300 mM NaCl and 10 mM imidazole. After the addition of lysozyme to 1 mg/ml, the cells were incubated on ice for 30 min before sonication (six times for 15 s). Then, the cell debris were removed by centrifugation (30 min, 10,000×*g*) and the LicA variants containing a histidine hexapeptide (His-tag) were further purified from the supernatant using Ni-NTA spin columns according to the protocol for native conditions (Qiagen). The active fractions were collected and purified to homogeneity on a Superdex200 column by FPLC (Amersham Biosciences). Depending on subsequent analysis of the enzyme, elution was performed with either a 50-mM sodium phosphate buffer (pH 7.7) or a 20-mM PIPES buffer (pH 7.0).

### Enzymatic Assays

Standard enzymatic assays were performed at 55 °C in 30 mM sodium phosphate buffer (pH 7.0) containing 0.1 mM of CaCl_2_ and barley β-glucan (final concentration, 0.4 %, *w*/*v*) as a substrate. The reducing sugars were detected by the dinitrosalicylic acid method, with glucose as the standard. One unit is defined as the amount of enzyme required to release 1 μmol of reducing sugars per minute. Temperature-induced inactivation was determined in 50 mM sodium phosphate (pH 7.7) and 1 mM CaCl_2_ by heating the purified enzyme (30 μg/ml) in small crimp-sealed vials, submerged in a water bath. During a time series (0–20 h), 20 μl aliquots were tested for remaining activity as described above. All activities were corrected for spontaneous hydrolysis in the absence of enzyme. In all cases, the standard errors of the enzymatic activity measurements (performed in duplicate) were <10 %.

### Thrombin Digestion

To linearize the circular proteins containing a thrombin recognition site, incubation with thrombin (Sigma) was performed overnight at 22 °C. A ratio of 1 U of thrombin per 100 μg of protein was used.

### Fluorescence Emission Spectroscopy

For fluorescence experiments, LicA variant proteins were purified with 20 mM PIPES buffer (pH 7.0) as the eluent in the final step on the Superdex200 column. In 10-mm Quartz SUPRASIL precision cells (Hellma), 20 mM PIPES (pH 7.0) was mixed with the protein solution to a final concentration of 15 μg/ml with a final volume of 3 ml. When indicated, 1 mM CaCl_2_ or 1 mM EDTA was added. Fluorescence emission was measured in the temperature range 30–90 °C, with a scan rate of 0.5 °C/min, by a Varian Cary Eclipse spectrophotometer. The emission spectra were recorded in the range 300–400 nm upon excitation of the tryptophans at 295 nm; the excitation and emission slit widths were set at 10 nm and the photomultiplier at 610 V. All spectra were corrected for the background emission of water. The emission spectra of samples containing 1 mM of either CaCl_2_ or EDTA were corrected using buffer baselines acquired at the same conditions.

### Differential Scanning Calorimetry

Temperature-controlled calorimetric studies of 0.3 mg/ml linear LicA-L1 and circular LicA-C1 were carried out in a VP-DSC calorimeter (MicroCal Inc., Northampton, MA) using as reference the appropriate buffer. All samples were degassed for 15 min prior to loading the cells, and the enzyme solution was kept under 1.5 bar pressure to avoid boiling of the sample. The temperature increased with a heat rate of 0.5 °C/min.

## Results and Discussion

### Engineering a Circular LicA

To produce a circular enzyme, we used a trans-splicing intein to ligate protein backbones. The intein PI-*Pfu*I from *P. furiosus* has been demonstrated to perform this cyclization in *E. coli* [24]; therefore, it was selected for the present study. In a two-step PCR reaction, we connected the C-terminal part of the intein (residues 161–454) to the N-terminus of the mature endo-1,3-1,4-β-glucanase and the N-terminal part (residues 1–160) to the C-terminus of the endo-1,3-1,4-β-glucanase (Fig. 3a). After cloning in pET24d, we produced a chimeric gene of 2,058 bp coding for a precursor protein with a total length of 686 amino acids (79.4 kDa), which did not contain the signal sequence of the wild-type LicA to ensure that splicing occurred intracellularly.

To facilitate purification of the LicA variants, a His-tag was introduced at the N-terminus of the glucanase. Furthermore, the connecting loop contained a thrombin recognition site (LVPRGT) to enable subsequent linearization and production of the LicA-L1 linear glucanase by thrombin cleavage (Fig. 3c).

**Fig. 4:**
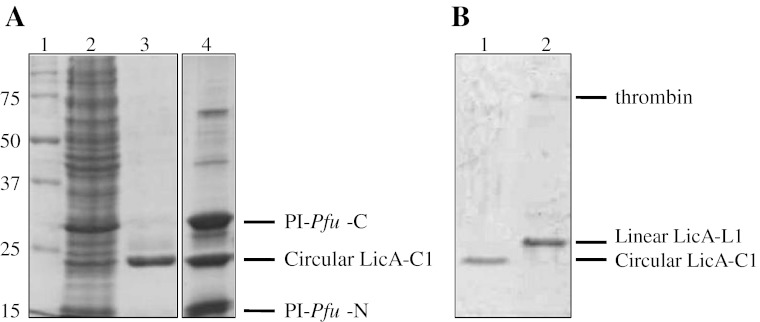
SDS-PAGE analysis of expressed and purified circular and linear LicA. **a** *Lane 1*, Molecular weight marker with corresponding sizes shown on the *left* (in kilodaltons). *Lane 2*, Cell-free extract of *E. coli* BL21(DE3) expressing construct I. *Lane 3*, Circular LicA-C1 after purification on a Ni-NTA column. *Lane 4*, Circular LicA-C1_control_ after purification on a Ni-NTA column. PI-*Pfu*I-C and PI-*Pfu*I-N denote the C- and N-terminal part, respectively, of the intein. **b** LicA-C1 after purification before (*lane 1*) and after (*lane 2*) treatment with thrombin

Upon the addition of thrombin to the purified spliced LicA-C1 protein and overnight incubation, SDS-PAGE analysis showed a migration behavior of the thrombin-digested sample (linear LicA-L1; Fig. 4b, lane 2) which was different from that of the untreated LicA-C1 protein (Fig. 4b, lane 1). The non-digested circular LicA-C1 protein migrates faster, as expected for a circular form. It is anticipated that in denaturing conditions, the circular enzyme is likely to retain a more compact (less unfolded) conformation compared to that of the thrombin-linearized form of the enzyme. This phenomenon has been reported previously for other circular and linearized polypeptides [22, 24, 25]. After thrombin treatment, the linear LicA-L1 consists of 224 amino acids, whereas the circular protein LicA-C1 has a length of 229 amino acids (Fig. 3b, c).

After purification, LicA-L1 has a specific activity of 2.7 U/mg against barley-β-glucan, which is similar to that of wild-type LicA expressed with the signal sequence [36]. This indicates that both the loss of the signal peptide (29 amino acids) and the addition of the His-tag (Fig. 3b) to the peptide backbone do not affect the specific activity of LicA.

After purification, SDS-PAGE gel analysis of cell-free extracts from *E. coli* BL21(DE3) cells overproducing LicA-C1 showed a single fragment of size 27 kDa corresponding to the molecular weight of the mature circular LicA-C1 with the C- and N-terminal parts of the intein spliced out (Fig. 4a, lane 3). Because neither of the intein fragments contains a His-tag, they will be lost during affinity chromatography. This allows for a convenient one-step purification of the produced circular LicAs.

As a splicing control, an additional construct was made, i.e., LicA-C1_control_, which is identical to LicA-C1, except that a second His-tag was introduced to the N-terminal part of the intein. In this case, SDS-PAGE analysis of the purified proteins revealed three bands with sizes of 35, 27, and 19 kDa (Fig. 4a, lane 4). The sizes correspond to the expected molecular weights of the LicA-C1 and of the C- and N-terminal parts of the intein. This suggests that the splicing reaction which occurred inside the cells leads to three separate polypeptide fragments. Moreover, the co-purification on the Ni-NTA column shows that the C-terminal part of the intein, lacking a His-tag, is associated with its N-terminal part, which is necessary for the splicing reaction. A similar phenomenon was observed by Iwai et al. [24] who also reported the presence of an unprocessed 80-kDa product. The absence of such an unprocessed form in our system indicates the efficiency of the present approach.

### Different Loop Designs

The cell-free extracts containing the circular protein LicA-C1 showed hydrolytic activity on barley-β-glucan, reflecting its proper folding. After purification, the specific activity of the circular enzyme LicA-C1 was calculated to be approximately two times higher compared to that of the linear LicA-L1 glucanase (Fig. 3c). To investigate the effect of the introduced loop on LicA’s biological activity, a series of constructs with different lengths and composition were designed. Shorter loops were constructed either by deletion of three N-terminal residues (QTG), two C-terminal residues (KR), the thrombin recognition site (LVPRGT), or a combination thereof, resulting in linking loops consisting of 9–20 amino acid residues (Fig. 3c). The six histidine residues were always maintained to facilitate purification. Based on SDS-PAGE analysis and biological activity assays of Ni-NTA-purified samples of the complete set of clones, it was concluded that not all circular variants were functionally produced. In the cases of LicA-C3, LicA-C4, and LicA-C6, no LicA protein was observed in the soluble fraction of *E. coli* lysates, and no activity was detected (Fig. 3c).

The two variants with the shortest loop (i.e., LicA-C3 and LicA-C6) were inactive, whereas the ones with the longest loop (LicA-C1 and LicA-C5) show significant hydrolytic activity (Fig. 3c). Circular LicAs with medium-sized loops presented an active (i.e., LicA-C2) and an inactive (i.e., LicA-C4) variant, which suggests that the loop length is not the only factor affecting biological function and that the C-terminal residues (KR) could be important for the stability of the circular enzyme. Further work will be required to elucidate the role of the C-terminal residues on the LicAs enzymatic activity. Removal of the N-terminal QTG or the thrombin recognition site has not any effect on the activity. Overall, it is concluded that both the length of the loop and the nature of amino acid residues in the region adjacent to the C-terminus determine the efficiency of intein processing which is required to generate active circular LicAs.

**Fig. 5:**
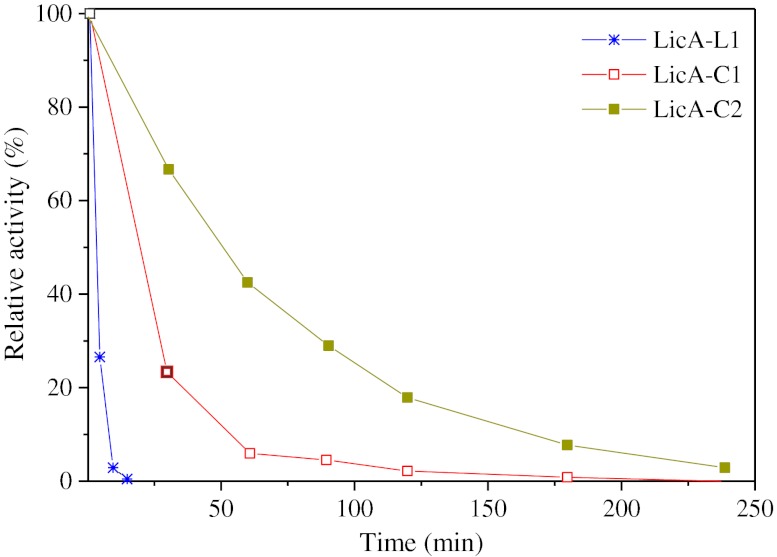
Temperature-induced inactivation. Residual activity of the linear LicA-L1 and the circular LicA-C1 and LicA-C2 glucanases when incubated at 65 °C at a concentration of 30 μg/ml in 50 mM sodium phosphate buffer (pH 7.7) with added 1 mM CaCl_2_. Activities are expressed relative to the activity of each enzyme measured at *t* = 0, which was taken as 100 %. Data represent the average (*n* = 2)

The variant with the shortest loop that was still active, LicA-C2, was compared with the long-loop variant, LicA-C1, with respect to temperature of optimal activity and thermal stability. The optimal temperature of hydrolytic activity for both spliced proteins was determined to be 56 °C, which is identical to the 56 °C optimum of the linear enzyme and to the 55 °C optimum of the wild-type enzyme [36]. However, the circular variants are more stable than the linear enzyme. Upon incubation at 65 °C, LicA-C1 inactivated sixfold slower than LicA-L1, whereas LicA-C2, the other circular variant with the shorter linker loop (Fig. 3c), showed an inactivation half-life time of 50 min at 65 °C, which implies a 16-fold increase compared to the inactivation half-life of the linear enzyme (Fig. 5).

The difference in stability between the two circular variants is significant. LicA-C2 differs from LicA-C1 by the deletion of six amino acids, corresponding to the thrombin recognition site from the connecting loop (Fig. 3c). The shorter loop of LicA-C2 might result in a more stable protein because it is more compact, whereas it is likely that the longer loop of LicA-C1 is more flexible, which renders the protein more susceptible to unfolding at elevated temperatures. Solving the three-dimensional structures of LicA-C1 and LicA-C2 would be required to reveal interactions between residues in the loop and other residues that might contribute to the stability of the circular enzymes.

Fluorescence spectroscopy after excitation at 295 nm showed similar emission spectra for the linear LicA-L1 and the circular LicA-C1, LicA-C2, and LicA-C5 proteins (not shown), confirming their correct folding. From these results, we conclude that circular LicA was expressed, excised, and ligated successfully to acquire its designed circular backbone. Furthermore, the increased activities of the circular enzyme variants LicA-C1, LicA-C2, and LicA-C5 compared to the linear LicA-L1 suggest that the introduced junction and loop do not result in activity loss of the enzyme. Earlier studies have shown that circular permutations in the compact jelly roll domain of LicA are tolerated and did not result in significant changes in the enzymatic activity or tertiary structure [37, 38]. Therein, it was shown that the direct peptide bond linking of the N- and C-termini, without the addition of extra residues, did not introduce strain into the molecule. However, in those studies, new N- and C-termini were introduced in another loop of the glucanase structure, and as such there was still an open-chain (linear) structure, which might enable the release of the strain. In our study, a covalently closed circular polypeptide chain was generated, resulting in increased specific enzymatic activity without introducing a severe strain.

### Thermal Stability of Circular LicA

**Fig. 6:**
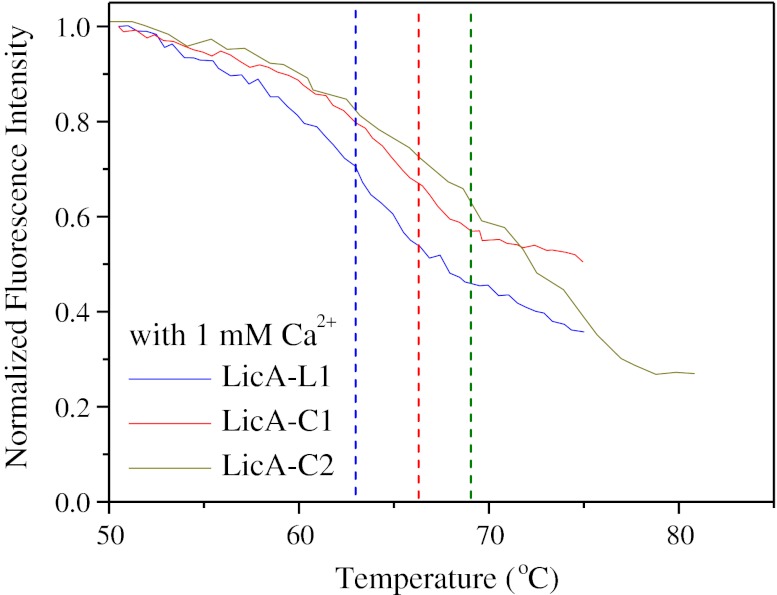
Temperature-induced unfolding. Fluorescence spectroscopy measurements of the fraction of folded linear LicA-L1 and circular LicA-C1 and LicA-C2 glucanase upon increasing temperature in the presence of 1 mM CaCl_2_. Fraction values were calculated by normalizing the fluorescence emission intensity data at 345 nm to the respective value at 30 °C for each enzyme variant. Excitation was at 295 nm. The transition temperature of unfolding was determined upon differentiation of the fluorescence intensity vs. temperature data and calculation of the minimum using the differentiate function or the sigmoidal fitting of Microcal Origin™ software

Temperature-induced unfolding was monitored by fluorescence spectroscopy to determine the transition midpoint and study the effect of cyclization on protein stability. LicA contains eight tryptophan residues randomly distributed over the protein backbone which were used as intrinsic probes for fluorescence emission studies. The fluorescence intensity of the linear and circular LicAs was monitored at 345 nm upon excitation at 295 nm as a function of temperature (Fig. 6). Upon increasing the temperature of the enzyme solution, the fluorescence intensity decreased and a red shift of the maximum fluorescence intensity was observed. The decrease in fluorescence was due to the unfolding of the tertiary structure of the enzyme. Data analysis showed that there is a clear effect of circularization toward stabilization of LicA. The circular glucanases unfolded at higher temperatures compared to the linear enzyme. In the presence of 1 mM CaCl_2_, the observed transition for LicA-L1 was determined to be at 62.6 ± 0.2 °C. The respective transitions for the circular LicA-C1 and LicA-C2 were 66.4 ± 0.1 and 69.0 ± 0.2 °C, respectively (Fig. 6).

**Fig. 7:**
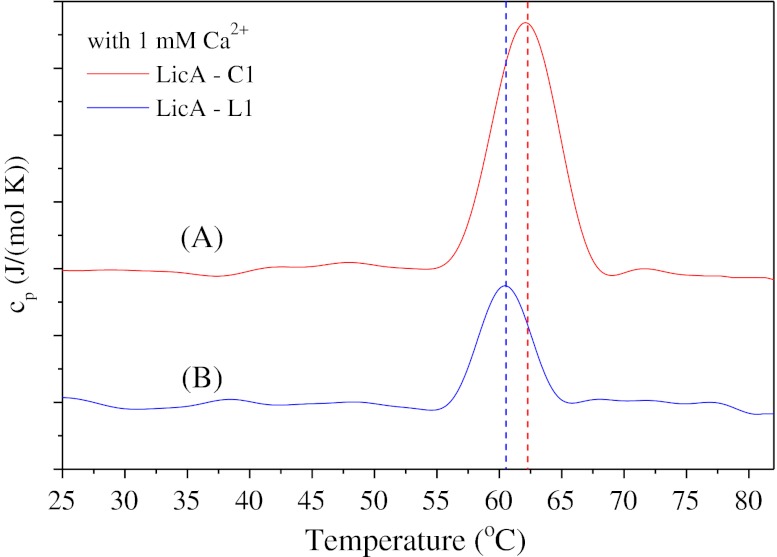
Temperature-induced unfolding. Differential scanning thermograms of the circular LicA-C1 (*A*) and the linear LicA-L1 (*B*). Protein concentration is 0.3 mg/ml in 20 mM PIPES buffer, pH 7. Graphs were vertically shifted for comparison. *Vertical dotted lines* show the shift of the denaturation temperature

Furthermore, the thermal unfolding of the linear LicA-L1 and circular LicA-C1 was studied by calorimetry. Differential scanning calorimetry (DSC) studies revealed a single transition peak in both cases. The denaturation temperatures, *T*_d_, were found to be 60.2 and 62.4 °C for LicA-L1 and LicA-C1, respectively (Fig. 7). These results are in good agreement with the transitions observed from monitoring the fluorescence intensity as a function of temperature at the same buffer conditions. Varying the scanning rate between 0.25 and 1.5 °C/min did not affect the *T*_d_ or the shape of the endothermal peak. As judged by the shape of the post-transition peak, it is suggested that heat denaturation results in protein aggregation. Furthermore, after cooling the samples to room temperature, subsequent heating up did not reveal a denaturation peak, which suggests irreversible unfolding. The difference in the transition temperature observed by fluorescence spectroscopy and DSC was expected because fluorescence spectroscopy monitors changes in the Trp microenvironment and therefore may be considered as a more sensitive method to monitor structural changes compared to calorimetry, in which a transition is recorded only when the protein unfolds locally or globally.

These results are in line with the results from comparing the thermal inactivation of LicA-C1 with that of LicA-L1 (Fig. 5). Incubation at 65 °C caused the linear enzyme to inactivate very rapidly (*t*_1/2_ = 3 min); complete inactivation occurred within 15 min. The circular LicA-C1, however, retained half of its initial activity even after 20 min (Fig. 5) at 65 °C, i.e., a sixfold increase compared to the linear LicA-L1. Taken together, the results of DSC, fluorescence spectroscopy, and biological activity tests suggest that backbone cyclization increases the internal stability of LicA-C1.

### The Effect of Calcium on the Thermal Stability of Circular LicA

**Fig. 8:**
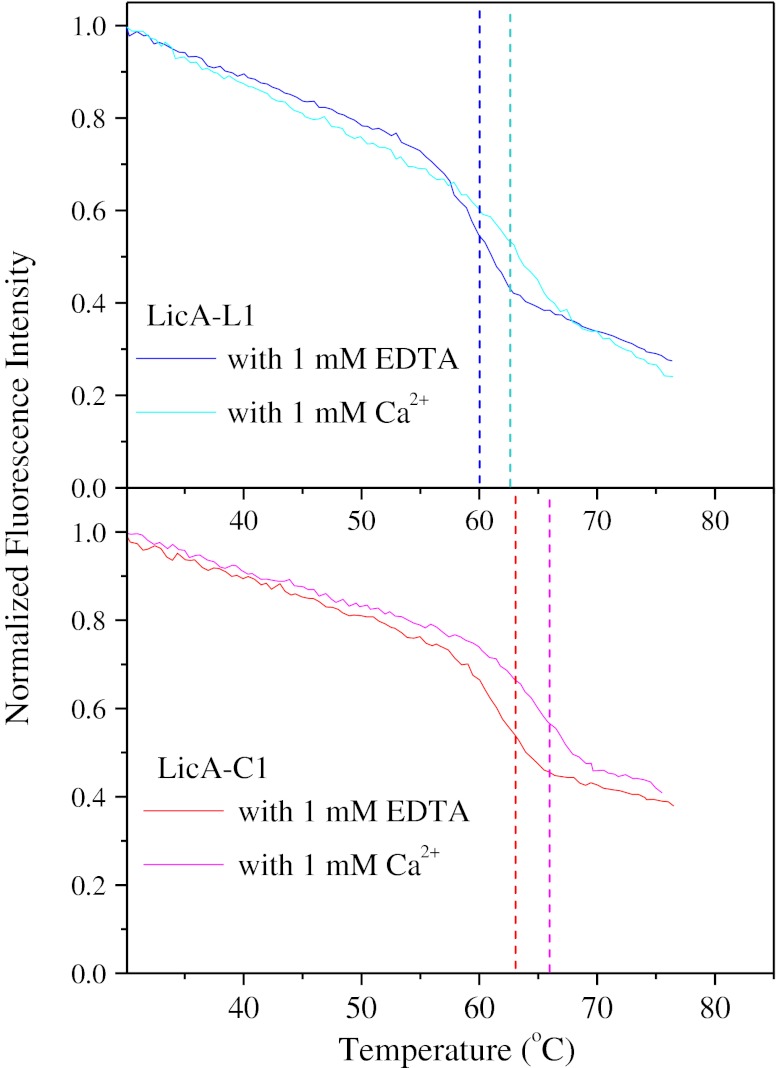
Temperature-induced unfolding measured by fluorescence spectroscopy of the linear and circular LicA with 1 mM CaCl_2_ or 1 mM EDTA. Fraction values were calculated by normalizing the fluorescence emission intensity data at 345 nm to the respective value at 30 °C. Excitation was at 295 nm. The transition temperature of unfolding was determined upon differentiation of the fluorescence intensity vs. temperature data and calculation of the minimum using the differentiation function of Microcal Origin™ software

The effect of calcium on the thermal stability of the linear and circular LicAs was examined by fluorescence spectroscopy. The addition of 1 mM EDTA caused the linear enzyme to denature at 59.8 ± 0.2 °C, which is lower compared to the melting temperature of 62.6 ± 0.2 °C that was observed in the presence of calcium (Figs. 6 and 8). This finding confirmed the stabilizing effect of calcium on LicAs, as reported previously for other members of the GH-16 family [39, 40]. In the presence of 1 mM EDTA, the circular LicA-C1 showed a melting temperature of 62.8 ± 0.2 °C, a decrease of 3.6 °C compared to the transition of 66.4 ± 0.1 °C which was observed in the presence of 1 mM CaCl_2_ (Figs. 6 and 8).

The apparent melting temperatures determined for the linear and circular enzymes agree with the deactivation of the glucanases at 65 °C (Fig. 5). In the presence of calcium ions, the melting temperature of the linear enzyme is at 62.6 °C; therefore, at 65 °C, in which the activity tests were performed, denaturation occurs rapidly. However, 65 °C is below the circular enzyme’s denaturation temperature (i.e., 66.4 °C for LicA-C1), resulting in a slower inactivation process.

In summary, the endo-1,3-1,4-β-glucanase from *B. licheniformis* was successfully circularized using the cyclization approach based on circular permutation of a precursor protein flanked by two intein domains. The circular variants presented activity properties which were up to three times higher than that of the linear enzyme; moreover, they were significantly more stable at elevated temperatures. It is anticipated that apart from the stabilization effect of cyclization against heat and chemical treatment, circular enzyme variants would be more resistant toward exo-proteases compared to respective linear enzyme, as shown previously [17]. Successful implementation of thermozymes to numerous applications in biotechnology and biocatalysis depends on their ability to retain biological activity for prolonged periods of time upon exposure to high-temperature conditions. Although the design of the connecting loop will differ depending on the specific protein, the results presented in this study suggest that cyclization may be an effective tool to stabilize proteins and enzymes.

## Abbreviations

LicA: Endo-β-1,3-1,4-glucanase
DSC: Differential scanning calorimetry
